# Evaluation of the Persian Transcript of the COPD Assessment Test in the Measurement of COPD Health Status in Iranian COPD Patients

**DOI:** 10.5539/gjhs.v8n5p225

**Published:** 2015-10-20

**Authors:** Alireza Azargoon, Mohammadreza Gholami, Ali Farhadi, Maryam Hadi Chegni, Abolfazl Zendedel

**Affiliations:** 1 School of Medicine, Lorestan University of Medical Sciences, Khorramabad, Iran

**Keywords:** COPD Assessment Test, chronic obstructive lung disease, Global initiative lung disease

## Abstract

**Aim:**

Chronic obstructive pulmonary disease is a completely irreversible obstructive airway disease. The COPD assessment test (CAT) is one of the standard methods for the clinical assessment of the disease, which is translated into Persian. This study investigated the reliability of the test and its relationship with the severity of the disease.

**Methods:**

In this cross-sectional study, 120 patients filled out the Persian transcript of the test. After two weeks, the patients filled out the CAT test again. Obstruction severity was determined for all the patients using spirometry, and the patients were categorized into four groups according to the Global Initiative for Chronic Obstructive Lung Disease criteria. The relationship between the test scores and the disease severity wan validated.

**Results:**

The mean age of the patients was 51.5 years. The Cronbach’s alpha coefficient of the Persian transcript of the test was 0.872 in the first time, and 0.885 in the second time. Intragroup reliability, test re-test and intragroup correlations were significant for all the questions (<0.001). The relationship between the test mean score and obstruction severity was significant, and the correlation between disease categorization in accordance with obstruction severity and categorization according to the test score was significant as well.

**Conclusion:**

The Persian transcript of the assessment test for COPD was reliable and is directly related to the disease severity according to airflow limitation.

## 1. Introduction

Chronic obstructive pulmonary diseases (COPDs) are the most common diseases in the community and one of the main causes of morbidity and mortality in the elderly (Andreoil et al., 2007). COPD is a term that includes a group of lung disorders (Wedzicha, 2011; Valero et al., 2009). COPDs are known with pulmonary alveolar destruction and grow up with a cough, wheeze, chronic sputum and narrowing of the respiratory bronchioles (Wedzicha, 2011; Valero et al., 2009). COPD diseases, including emphysema, chronic bronchitis, bronchiolitis and chronic lung disease, are also common clinical, radiographic, and physiologic protests, and are more often found in patients simultaneously (Andreoil et al., 2007; Wedzicha, 2011; Valero et al., 2009). Other obstructive lung diseases such as asthma, cystic fibrosis and bronchiectasis are distinct conditions in terms of disease onset, symptoms (Andreoil et al., 2007; Wedzicha, 2011; Valero et al., 2009; Hernandez et al., 2013; Zendedel et al., 2015). COPD prevalence is 6-10%, approximately 300 million people in the world, in the adult population (Andreoil et al., 2007; Wedzicha, 2011; Valero et al., 2009). COPD after cardiovascular disease, cancer and cerebrovascular diseases are the fourth cause of death in America. To achieve good control of the disease requires patient participation in the treatment process and helps the doctor’s understanding of their disease (Jones et al., 2011). A number of studies have shown that there are differences between the expected health and the life quality of the patients (Glaab et al., 2012). Usually patients cannot properly describe the severity of their disease. On the other hand, specialists do not have enough time to collect this information, as a result of the way that physicians understand the clinical status of the patient (Glaab et al., 2012). The most effective way is using a short questionnaire that is fast, reliable and standardized. In this context, researchers have designed patient-based questionnaires to assess the symptoms and the patient’s health status. Standard questionnaires such as St. Gorge Respiratory Questionnaire (SGRQ), Clinical Questionnaire COPD and Chronic Respiratory Disease Questionnaire have numerous and complex questions, so that answering them is boring and their interpretation by the patient at the bedside is difficult (Marchand & Maury, 2012). The COPD Assessment Test (CAT) was designed by Paul Jones in 2009, and is a new and simple method for measuring the quality of health care in COPD patients (Jones et al., 2009). The CAT was designed by a group that included lung specialists, general practitioners and representatives of patients. Several studies have been done to evaluate the CAT in Europe, Asia and America (Glaab et al., 2012; Jones et al., 2009). The CAT questionnaire has 8 questions, each having a 0-5 score (total range 0-40) with a score >10 being abnormal. The CAT is easier for patients and more reliable in comparison with FEV_1_. The questionnaire is appropriate for all the patients with a diagnosis of COPD and of all intensities (based on the classification of GOLD). This questionnaire is not a diagnostic tool and cannot be used instead of spirometry. The questionnaire was translated into 62 different languages and validated in different populations with different languages, including Arabic, Chinese and Japanese, and the validity and reliability of the test have been proven in these studies (Glaab et al., 2012). The purpose of this study was to evaluate the reliability of the Persian version of this questionnaire and its relationship with pulmonary function (patient spirometry parameters).

## 2. Method

This cross-sectional study was performed in Khorramabad city in 2014. The patients selected were those with COPD referred to Ashayer and Rahimi hospitals of Khorramabad city. The inclusion criteria of the study included a history of smoking, symptoms of cough and sputum production for at least three months in two consecutive years, evidence of pulmonary emphysema on CT scan, and evidence of obstructive pulmonary disease on spirometry according the COPD guidelines. The spirometry was performed for the selected patients, and FEV1 and FVC values were recorded and their relationship was calculated. The CAT questionnaire was translated by GlaxoSmithKline into 62 languages including Persian. The questionnaire has eight questions, including symptoms of cough, sputum, feeling of heaviness in the chest, shortness of breath during activity, restrictions on activities, sleep, energy levels of the patient, and the patient’s sense of security when leaving home. Each question has 6 points (0-5), and the total points ranges from zero to 40. According the total points, the patients were placed into four groups (Table 1). The patients were guided about how to respond the questions. To assess the validity, there was another response to the questionnaire two weeks later. To evaluate the relationship between the Persian version of the questionnaire and lung function, spirometry using SpiroLab3 was performed and the values of FEV1 and FVC along with the ratio of these two criteria were recorded. The patients were divided into four groups according to the COPD severity on the basis of GOLD spirometry (Table 1).

**Table 1:** The mean, standard deviation and Cronbach’s alpha of the CAT questions

Question	mean	Standard Deviation	Cronbach’s alpha
**Cough**	3.08	1.32	0.873
**Sputum**	3.05	1.29	0.863
**Chest heaviness**	3.41	1.35	0.849
**Shortness of breath climbing stairs**	3.35	1.37	0.847
**Restrictions on Activities**	2.27	1.22	0.843
**Leaving home safely**	1.89	1.14	0.856
**Sleep**	2.50	1.56	0.866
**The patient’s Energy**	2.45	1.14	0.853

### 2.1 Statistical Analysis

Statistical analyses were performed using the SPSS software. T-test analysis of variance and Pearson’s correlation coefficient were used to assess the relationship between the parametric variables, with a p≤0.001 considered significant.

## 3. Results

In this study, 120 patients were studied. The mean patient age was 51.5 years, and 74.2% of the population were male. Thirty-three percent of the patients had only smoking and other risk factors include burning wood, dust, tobacco production and opium consumption. The CAT questionnaire reliability was defined using Cronbach’s alpha and the intragroup correlation in two stages. Cronbach’s alpha was 0.872 in the first stage, and 0.885 in the second stage. The results of the intragroup correlation in the first stage and the second stage were 0.87 and 0.88, respectively. For validity, the coefficient of correlation and Kappa were used between the disease and the CAT, and the scores were classified according to GOLD criteria. The CAT scores, Table 1, were divided into three groups, the patients were divided into four groups on the basis of GOLD spirometry, and the correlation of the results was evaluated. The relationship between the scores of the CAT and COPD disease is presented according to the GOLD criteria (Table 2). According to the GOLD criteria, 57 patients were in Stage 1, 34 patients in Stage 2, 27 patients in Stage 3, and 14 patients in Stage 4. The CAT questionnaire results for the 57 patients with Stage 1, according to the GOLD criteria, showed that 28 and 21 patients had moderate impact and high impact, respectively. The CAT questionnaire results for the 34 patients with stage 2, according to the GOLD criteria, showed that 15 and 17 patients had moderate impact and high impact, respectively. Stage 3 of the disease included 15 cases, with 9 cases in the category of high impact. Finally, 14 patients were in Stage 4 of the disease, with 8 in the category of very high impact. The above-mentioned results showed a correlation between the scores of the CAT, and COPD disease according to the GOLD criteria.

**Table 2:** Impact level of COPD on health status and GOLD spirometry criteria

GOLD spirometry criteria		Impact level of COPD on health status	
Stage 1	FEV1> 80%	**Low impact**	**Score < 10**
(mild disease)	FEV1/FVC< 70%		
Stage 2	FEV1: 50 – 80%	**Mod impact**	**Score : 10 – 20**
(moderate disease)	FEV1/FVC< 70%		
Stage 3	FEV1: 30 – 49%	**High impact**	**Score: 21 – 30**
(sever disease)	FEV1/FVC< 70%		
Stage 4	FEV1< 30%	**Very high impact**	**Score > 30**
(very sever disease)			

**Table 3:** The relationship between the scores of the CAT, and COPD disease according to the GOLD criteria

GOLD	CAT				Tot

	Low impact	Mod impact	High impact	Very high impact	
Stage 1	6	28	21	2	**57**
Stage 2	1	15	17	1	**34**
Stage 3	1	2	9	3	**15**
Stage 4	0	2	4	8	**14**
Total	8	47	51	14	**120**
Value of Kappa	**136**				
Significant level	**0.001**				

## 4. Discussion

Many questionnaires have been designed to assess the severity of lung disease. The questionnaires have been designed on the basis of the clinical symptoms of the disease. The questionnaires have been translated into other languages, and validation studies have been designed for the assessment of translated versions. The CAT is a new and simple method to evaluate the quality of health in patients with COPD. This study showed that the Persian transcript of the COPD assessment test was reliable and is directly related to disease severity according to air way obstruction. The CAT was known as a reliable test to assess asthma control (Pothirat et al., 2014). Mackay et al. conducted a study to assess the usefulness of the CAT in 161 patients with the exacerbations of COPD. The patients were studied in the stable phase of the disease exacerbation and remission period of one year, and completed the CAT questionnaire. The CAT in periods of exacerbation reflects the deterioration of lung function (Mackay et al., 2012). The Persian translation of the CAT with internal reliability and validity has a direct relationship with the severity of airflow obstruction and the severity of the patient’s smoking (Sigari and Ghafori, 2012). The results of this study were consistent with the results obtained by Paul Jones. The Persian translation also shows the high internal correlation with the results obtained from the study of the original and translated versions in Arabic and Chinese (Mackay et al., 2012). Nagata et al. reported that the CAT questionnaire is valid for evaluating patients with COPD (Nagata et al., 2012). The Chinese version of the CAT can be a valid and reliable standardized way to assess the health status of COPD patients, and can be used instead of SGRQ (Wiklund et al., 2010). Recently, Pothirat et al. showed that the Thai translation of the CAT has a valid, acceptable and standard form for COPD health measurement (Pothirat et al., 2014). The CAT is not a diagnostic test, but there is a correlation between airflow obstruction and the CAT questionnaire (Ghobadi et al., 2012). The CAT can be a good way to evaluate a patient with a relatively controlled or uncontrolled asthma according to the GINA criteria (Yawn et al., 2006). The Arabic version of the CAT used to assess COPD disease is valid and reliable (Al-Moamary et al., 2011). Chetta et al. proposed that in the future, the CAT questionnaire can be used for the patient’s assessment and improve communication between specialists and patients (Chetta et al., 2012). Lee et al. showed that the CAT questionnaire is a simple, reliable and cost-effective method for the assessment of COPD exacerbations in high-risk patients (Lee et al., 2014). During chronic obstructive pulmonary disease exacerbations, the CAT questionnaire can be useful and reliable in patients’ overall health status (Feliz-Rodriguez et al., 2013). In COPD patients, the CAT questionnaire is a complementary tool for spirometry results and helps specialists to treat COPD patients (Papaioannou et al., 2014). Research showed that the Italian validated version of the CAT questionnaire is a good, valid and reliable instrument for the assessment of COPD disease. Recently, research has showed that the CAT questionnaire is an international cross-cultural assessment instrument that helps doctor monitor patients’ health status and facilitates communication between physicians and patients. The CAT questionnaire is not a diagnostic test for lung function, but it is an instrument to explain the status of COPD patients for specialists that provide clinical and therapeutic monitoring of COPD patients. The CAT is a good tool for following up COPD patients (Pinto et al., 2014). The results of this study were consistent with the Thai translation of the CAT. The Persian version can be used to assess the health status of COPD patients. Taken together, COPD diagnostic criteria with spirometry are correlated with clinical signs in the diagnosis of this disease. Therefore, this questionnaire should be used only to provide diagnoses and to assess treatment.
